# Stable self-serving personality traits in recreational and dependent cocaine users

**DOI:** 10.1371/journal.pone.0172853

**Published:** 2017-03-02

**Authors:** Boris B. Quednow, Lea M. Hulka, Katrin H. Preller, Markus R. Baumgartner, Christoph Eisenegger, Matthias Vonmoos

**Affiliations:** 1 Experimental and Clinical Pharmacopsychology, Department of Psychiatry, Psychotherapy, and Psychosomatics, Psychiatric Hospital, University of Zurich, Zurich, Switzerland; 2 Zurich Institute of Forensic Medicine, Center for Forensic Hair Analysis, University of Zurich, Zurich, Switzerland; 3 Neuropsychopharmacology and Biopsychology Unit, Department of Basic Psychological Research and Research Methods, Faculty of Psychology, University of Vienna, Vienna, Austria; Radboud University Medical Centre, NETHERLANDS

## Abstract

Chronic cocaine use has been associated with impairments in social cognition, self-serving and antisocial behavior, and socially relevant personality disorders (PD). Despite the apparent relationship between Machiavellianism and stimulant use, no study has explicitly examined this personality concept in cocaine users so far. In the frame of the longitudinal Zurich Cocaine Cognition Study, the *Machiavellianism Questionnaire* (MACH-IV) was assessed in 68 recreational and 30 dependent cocaine users as well as in 68 psychostimulant-naïve controls at baseline. Additionally, three closely related personality dimensions from the *Temperament and Character Inventory* (TCI)–cooperativeness, (social) reward dependence, and self-directedness–and the screening questionnaire of the *Structured Clinical Interview for DSM-IV Axis II Personality Disorders* (SCID-II) were acquired. At the one-year follow-up, 57 cocaine users and 48 controls were reassessed with the MACH-IV. Finally, MACH-IV scores were correlated with measures of social cognition and interaction (cognitive/emotional empathy, Theory-of-Mind, prosocial behavior) and with SCID-II PD scores assessed at baseline. Both recreational and dependent cocaine users showed significantly higher Machiavellianism than controls, while dependent cocaine users additionally displayed significantly lower levels of TCI cooperativeness and self-directedness. During the one-year interval, MACH-IV scores showed high test-retest reliability and also the significant gap between cocaine users and controls remained. Moreover, in cocaine users, higher Machiavellianism correlated significantly with lower levels of cooperativeness and self-directedness, with less prosocial behavior, and with higher cluster B PD scores. However, Machiavellianism was not correlated with measures of cocaine use severity (r<-.15). Both recreational and dependent cocaine users display pronounced and stable Machiavellian personality traits. The lack of correlations with severity of cocaine use and its temporal stability indicates that a Machiavellian personality trait might represent a predisposition for cocaine use that potentially serves as a predictor for stimulant addiction.

## Introduction

Disturbed socio-cognitive abilities are important features of all substance use disorders, but they particularly affect stimulant-using populations [1, 2]. Accordingly, it has been proposed that social functioning and, thus, social beliefs and attitudes might play essential roles in the origin and course of stimulant addiction [3]. However, until recently, systematic investigations examining social characteristics and attitudes in stimulant users were scarce, although accumulating evidence suggests that, e.g., chronic cocaine use is associated with broad alterations in socio-cognitive functioning [4–6]. In particular, recent research showed that recreational cocaine users (RCU) exhibit deficits in emotional empathy and emotion recognition from prosody, whereas dependent cocaine users (DCU) show difficulties with emotional and mental perspective-taking (*Theory-of-Mind*) [5, 6]. Moreover, RCU and DCU show more self-serving behavior in social interaction paradigms, commit more criminal offenses, and have smaller social networks [4, 6]. In addition, dependent cocaine use is commonly associated with a variety of socially relevant personality disorders (PD), including antisocial and narcissistic PD [6, 7]. Altogether, these specific social characteristics of chronic cocaine users resemble the broad concept of Machiavellianism [8, 9], which describes a social attitude characterized by cynical beliefs, interpersonal manipulation, and pragmatic morality [10]. However, although social functioning was linked to drug addiction [3] and explicitly cocaine addiction has been associated with social isolation and the neglect of social rules [1], only a very limited number of studies have investigated the linkage between Machiavellianism and illegal substance use in general [11, 12], while no study has explicitly examined Machiavellian attitudes in cocaine users so far. Moreover, the currently closest link to Machiavellianism in cocaine users–the elevated self-serving behavior of RCU and DCU [4]–was established using money distribution games showing rather low test-retest reliabilities [13]. Accordingly, more reliable personality questionnaires might be an appropriate way to complement the existing findings on social behavior in cocaine users. Therefore, we aimed to investigate Machiavellianism in a large sample of RCU, DCU, and stimulant-naïve healthy controls (matched for age, sex, verbal IQ, and cigarette smoking) using the self-assessment Machiavellianism-questionnaire MACH-IV [10].

Based on previous studies mentioned above, we expected to find elevated Machiavellianism in both RCU and DCU. As Machiavellianism is generally considered to be a stable personality trait [8, 14], we further investigated the reliability and stability of the MACH-IV across an interval of one year and hypothesized that increased Machiavellianism in cocaine users remains stable over time. In addition, we examined three closely related [15] personality dimensions from Cloninger’s Temperament and Character Inventory (TCI, [16]) and expected less pronounced cooperativeness, reward dependence, and self-directedness in both cocaine users groups. Given that chronic cocaine users exhibit impaired socio-cognitive functioning and increased PD comorbidities [4–7] and that Machiavellianism has been negatively associated with socio-cognitive functioning [8] but positively linked to most PD [9], we investigated the association of Machiavellianism with socio-cognitive functioning and PD symptoms as well.

## Methods

### Participants

The present data were collected as part of the Zurich Cocaine Cognition Study (ZuCo^2^St), for which recruitment information has been previously published in detail [4, 6, 17]. The present study included a sample largely overlapping the studies of Vonmoos et al. [17, 18], consisting of 68 RCU, 30 DCU, and 68 healthy, stimulant-naïve controls at baseline. Specific inclusion criteria for the cocaine groups were cocaine use of at least 0.5g/month, with cocaine being the preferred illegal drug, and a current abstinence duration of <6 months. Cocaine dependence was diagnosed according to the Diagnostic and Statistical Manual-IV (*DSM-IV)* criteria [19], with only DCU fulfilling these criteria. Exclusion criteria for cocaine users were previous or present *DSM-IV* Axis-I adult psychiatric disorders other than cocaine, nicotine, and alcohol abuse/dependence; history of depression; and attention deficit hyperactivity disorder [20]. Moreover, the intake of opioids and a polytoxic drug use pattern according to *DSM-IV* were not permitted. Exclusion criteria for control subjects were any *DSM-IV* Axis-I psychiatric disorders, with the exception of nicotine dependence, and regular illegal drug use (lifetime use <15 occasions), with the exception of occasional cannabis use (≤1 occasion per day). For both groups, exclusion criteria were severe medical diseases, head injury or neurological disorders, and use of prescription psychotropic drugs. Urine screenings (semi-quantitative enzyme multiplied immunoassay method) and 6-month hair analyses (liquid chromatography tandem mass spectrometry) allowed the objective quantification of drug use (for details see [17, 18]).

For the longitudinal analysis, we re-invited the participants from the cross-sectional sample for a retest-session one year later. In the course of this, 48 psychostimulant-naïve controls and 57 cocaine users could be included in a subsequent one-year follow-up analysis (for recruitment and selection details see [21]. In this analysis, we applied the same group assignment as in two previous ZuCo^2^St subprojects (for selection details [21, 22]) following a combination of absolute (cocaine concentration of ≥±0.5 ng/mg, [23, 24]) and relative (minimal increase of 20% or a minimal decrease of 10% in cocaine_total_, [25]) changes in cocaine concentration in hair samples between baseline (t1) and a one-year follow-up (t2). According to these criteria, cocaine users were divided into three groups of similar size: 19 cocaine increasers (mean increase +30.4 ng/mg [+297%], range +0.5 to +268.5 ng/mg [+20% to +5374%], SD 61.9 ng/mg), 19 cocaine decreasers (mean decrease -10.6 ng/mg [-72%], range -116.9 to -0.6 ng/mg [-100% to -12%], SD 26.7 ng/mg), and 19 stable cocaine users who did not meet either of the former criteria (mean change -0.1ng/mg [-2%], range -1.9 to +0.5 ng/mg [-100% to +720%], SD 0.5 ng/mg).

At baseline and one-year follow-up, participants were asked to abstain from illegal substances for 72h and from alcohol for 24h before the testing session. The study was approved by the Cantonal Ethics Committee of Zurich. All participants provided written informed consent and were compensated for their participation.

### Procedure

Machiavellianism was assessed with a German version of the MACH-IV [10], which includes 20 items, with 7-point Likert-scales ranging from -3 to +3 (-3 = totally disagree, 0 = neutral, +3 = totally agree). In order to follow the original MACH-IV-instructions [10], we transformed the ratings into a 7-point Likert scale ranging from 1 to 7 (1 = totally disagree, 4 = neutral, 7 = totally agree) and added a constant of 20 to the total score (but not to the subscales). Cooperativeness, reward dependence, and self-directedness were measured with a German version of the 240-item TCI [16]. The Structured Clinical Interview for *DSM-IV* Axis-II Disorder questionnaire (SCID-II, [26]) was used to assess PD symptom severities. Social cognition and interaction were measured with the following tasks: Multifaceted Empathy Test (MET, [27]), Movie for the Assessment of Social Cognition (MASC, [28]), Reading the Mind in the Eyes Test (RMET, [29]), Distribution Game [30], and Dictator Game [31], which have been described in detail before [4, 6]. The Social Network Questionnaire (SNQ, [32]) was applied to estimate participants’ current social network sizes (see also [6]). Craving for cocaine was assessed by the brief version of the Cocaine Craving Questionnaire (CCQ, [33]). Self-reported cocaine use was assessed with the Interview for Psychotropic Drug Consumption [34].

### Statistical analysis

Statistical analyses were performed with IBM SPSS Statistics 22.0. Demographic and drug use data were analyzed by analyses of variance, Students t-tests and χ^2^-tests, where appropriate. MACH-IV and TCI scores were analyzed by analyses of covariance (ANCOVA), followed by Sidak-corrected post-hoc comparisons. Because previous findings suggest a positive link between advancing age and fairness in stimulant users [4] and to control for comprehension differences in the applied language-based questionnaires, age and verbal IQ were introduced as covariates. Verbal IQ was measured with the German vocabulary test *Mehrfachwahl-Wortschatz-Intelligenztest B* (MWT-B [35]), an estimate of premorbid verbal intelligence. The longitudinal analysis was carried out by a mixed design analyses of variance, again including the covariates age and verbal IQ. Pearson’s product-moment correlations were conducted across a combined cocaine user group (RCU+DCU). To test for potential differences between the correlation coefficients in controls and cocaine users, Fisher’s *z*-tests were performed [36]. The confirmatory statistical comparisons were carried out on a significance level of p < .05 (two-tailed). P-values in the Pearson’s product-moment correlations and Fisher’s *z*-tests were adjusted by Bonferroni correction. In the applied adjustment method, the p-values of the single correlations were multiplied by the number of analyzed variables per test to compare the results to the nominal significance level of 0.05 (TCI 16 variables, MET 3, MASC 4, RMET 1, Games 2, SNQ 1, SCID-II 16, cocaine use measures 10) [37].

## Results

### Demographic characteristics and drug use

As reported in Table 1 and also in previous papers using the same sample [17, 18], the three groups did not differ significantly at baseline regarding age, sex distribution, smoking status, socio-economic status, and verbal IQ. However, DCU showed fewer years of school education compared with RCU and controls. The RCU used cocaine regularly without fulfilling the *DSM-IV* criteria for dependence, whereas 30 DCU showed a comparatively higher weekly use of cocaine, higher cocaine hair concentration, and higher cumulative lifetime dose.

**Table 1:** Demographic data and drug use pattern (cross-sectional sample).

	Stimulant-naïve controls (n = 68)	Recreational cocaine users (n = 68)	Dependent cocaine users (n = 30)	F/χ^2^/T	df,df_err_	p	p, Sidak post-hoc		
							C-RCU	C-DCU	RCU-DCU
Age (y)	30.26 (9.25)	28.71 (6.19)	32.53 (8.96)	2.39^a^	2,163	.10	.60	.49	.09
Sex (f/m)	21 / 47	18 / 50	8 / 22	0.38^b^	2	.83			
Verbal IQ (MWT-B)	104.35 (9.68)	103.21 (9.58)	99.73 (9.11)	2.46^a^	2,163	.09	.86	.08	.27
School education (y)	10.66 (1.8)	10.50 (1.96)	9.48 (1.19)	4.82^a^	2,163	**.009**	.93	**.009**	**.03**
Smoking / Non-smoking	53 / 15	53 / 15	24 / 6	0.06^b^	2	.97			
Alcohol grams per week^k^	116.81 (122.65)	167.8 (117.47)	188.52 (260.55)	2.94^a^	2,163	.06	.16	.10	.90
Nicotine cigarettes per day^k^	9.33 (9.53)	11.70 (8.77)	15.71 (13.54)	4.21^a^	2,163	**.02**	.43	**.01**	.20
Cannabis grams per week^k^	0.45 (0.98)	0.86 (2.05)	1.24 (3.73)	1.53^a^	2,163	.22	.61	.26	.80
*Cocaine*									
Times per week^k^	-	1.07 (1.03)	2.88 (2.58)	4.97^c^	96	**< .001**			
Grams per week^k^	-	1.11 (1.41)	6.1 (8.74)	4.62^c^	96	**< .001**			
Years of use^d^	-	6.47 (3.99)	9.35 (6.51)	2.69^c^	96	**.008**			
Maximum dose (grams/day)^d^	-	3.46 (2.47)	9.42 (8.36)	5.39^c^	96	**< .001**			
Cumulative dose (grams)^d^	-	519.7 (751.2)	5500.9 (9635.2)	4.26^c^	96	**.008**			
Last consumption (days)	-	27.5 (37.6)	21.0 (33.6)	0.81^c^	96	.42			
Craving for cocaine (0–70)	-	19.0 (9.1)	20.3 (11.4)	0.60^c^	96	.55			
Urine toxicology (neg/pos)^g^	68 / 0	57 / 10	18 / 12	29.07^b^	2	**< .001**			
*Cocaine hair analysis*^e^									
Cocaine_total_ pg/mg^f^	-	3232 (5547)	28008 (40129)	4.98^c^	95	**< .001**			
Cocaine pg/mg	-	2624 (4575)	22374 (32509)	4.90^c^	95	**< .001**			
Benzoylecgonine pg/mg	-	546 (919)	5048 (7711)	4.73^c^	95	**< .001**			
Cocaethylene pg/mg	-	276 (316)	2006 (3656)	3.87^c^	95	**< .001**			
Norcocaine pg/mg	-	62 (101)	586 (758)	5.58^c^	95	**< .001**			
*Socioeconomic status*^h^^,^^i^				14.18^b^	10	.16			
0–15'000 CHF^j^	25 (36.8%)	18 (26.5%)	13 (43.3%)	3.12^b^	2	.21			
15'000–30'000 CHF^j^	16 (23.5%)	11 (16.2%)	10 (33.3%)	3.64^b^	2	.16			
30'000–60'000 CHF^j^	12 (17.6%)	20 (29.4%)	3 (10.0%)	5.53^b^	2	.06			
60'000–90'000 CHF^j^	11 (16.2%)	16 (23.5%)	2 (6.7%)	4.24^b^	2	.12			
90'000–120'000 CHF^j^	1 (1.5%)	2 (2.9%)	1 (3.3%)	0.45^b^	2	.80			
120'000 CHF and more^j^	3 (4.4%)	1 (1.5%)	1 (3.3%)	1.02^b^	2	.60			

Means and standard deviations. Significant p values (p < .05) are shown in bold. Sex and smoking are shown in frequency data.

^a^ ANOVA F-test (all groups).

^b^ χ^2^-test (all groups) for frequency data.

^c^ Independent t-test (cocaine user groups only).

^d^ Since first cocaine use.

^e^ Hair samples were voluntary and are deficient for 3 controls and 1 recreational cocaine user.

^f^ Cocaine_total_ (= Cocaine + Benzoylecgonine + Norcocaine) is a more robust procedure for discrimination between incorporation and contamination of hair [25].

^g^ Cut-off value for cocaine = 150 ng/ml. Urine toxicology test was deficient for 1 RCU.

^h^ Participants were asked how much money they had available over the past year (they had to choose one of the six status levels).

^i^ χ^2^-test over all six status levels and groups.

^j^ χ^2^-test per row.

^k^ Average use during the last 6 months.

In the longitudinal analysis the four investigated groups (controls, increasers, decreasers, stable users) did not significantly differ regarding age, sex distribution, verbal IQ, years of education, smoking status, and length of interval between the two study assessments (Table 2; for a more detailed sample description see [21, 22]).

**Table 2:** Demographic data and drug use pattern (longitudinal sample).

	Baseline (t1)							1-year follow-up (t2)^j^						
	Controls (n = 48)	Cocaine Increaser (n = 19)	Cocaine Decreaser (n = 19)	Stable cocaine users (n = 19)	F/χ^2^	df, df_err_	p	Controls (n = 48)	Cocaine Increaser (n = 19)	Cocaine Decreaser (n = 19)	Stable cocaine users (n = 19)	F/χ^2^	df, df_err_	p
Age (y)	30.3 (8.9)	31.5 (9.4)	31.4 (8.3)	27.0 (5.6)	1.20^a^	3,101	.31							
Sex (f/m)	16 / 32	3 / 16	5 / 14	8 / 11	3.49^b^	3	.32							
Verbal IQ (MWT-B)	107.6 (10.0)	102.9 (9.7)	103.8 (7.1)	104.5 (9.1)	1.57^a^	3,101	.20							
School education (y)	10.8 (1.8)	10.4 (1.8)	10.0 (1.5)	10.3 (1.6)	0.96^a^	3,101	.41							
Smoking / Non-smoking	37 / 11	14 / 5	14 / 5	14 / 5	0.16^b^	3	.98	40/8	15/4	13/6	15/4	1.83^b^	3	.61
Weeks between t1 & t2	58.2 (10.1)	59.3 (12.1)	61.9 (14.5)	64.8 (16.3)	1.37^a^	3,101	.26							
Alcohol grams/week^d^	119.9 (136.8)	169.4 (129.2)	155.3 (146.4)	132.3 (86.4)	0.81^a^	3,101	.49	104.3 (88.6)	259.7 (244.5)***	127.4 (141.4)°	146.7 (95.1)	5.74^a^	3,101	**.001**
Nicotine cigarettes/day^d^	8.7 (8.7)	12.8 (11.2)	9.5 (8.2)	12.2 (8.3)	1.32^a^	3,101	.27	8.2 (8.7)	13.4 (12.0)	8.2 (7.8)	12.7 (8.9)	2.23^a^	3,101	.09
Cannabis grams/week^d^	0.6 (1.6)	3.3 (8.9)	1.2 (2.3)	1.2 (2.6)	1.81^a^	3,101	.15	0.5 (1.6)	2.1 (4.6)	1.1 (2.7)	0.9 (1.6)	1.74^a^	3,101	.16
*Cocaine*														
Times/week^d^	-	1.6 (1.8)	1.0 (1.3)	0.6 (0.6)	2.51^c^	2,54	.09	-	1.1 (0.8)	0.3 (0.3)°°°	0.3 (0.2)°°°	15.6^c^	2,54	**< .001**
Grams/week^d^	-	2.0 (2.5)	1.7 (2.3)	0.7 (0.6)	2.26^c^	2,54	.11	-	1.6 (2.5)	0.4 (0.4)°	0.2 (0.3)°	5.39^c^	2,54	**.007**
Years of use^e^	-	7.0 (5.5)	8.2 (5.4)	5.4 (5.0)	1.40^c^	2,54	.25	-	8.9 (5.4)	9.7 (5.2)	6.3 (5.6)	2.09^c^	2,54	.13
Maximum dose (gr/d)^e^	-	4.7 (4.4)	5.9 (6.4)	3 (3.1)	1.78^c^	2,54	.18	-	3.7 (2.5)	3.1 (2.8)	1.7 (1.5)°	3.53^c^	2,54	**.04**
Cumulative dose (gr)^e^	-	1182 (1635)	3698 (8585)	394 (563)	2.21^c^	2,54	.12	-	91 (119)	49 (89)	18 (25)°	3.35^c^	2,54	**.04**
Last consumption (d)	-	18.5 (25.1)	20.8 (22.2)	42.2 (49.7)	2.72^c^	2,54	.08	-	7.0 (6.3)	81.4 (145.1)	58.2 (116.6)	2.38^c^	2,54	.10
Craving for cocaine	-	19.8 (9.5)	17.7 (7.2)	18.4 (7.7)	0.35^c^	2,54	.71	-	20.5 (10.8)	15.8 (6.2)	15.1 (7.7)	2.32^c^	2,54	.11
Urine toxicology (n/p)^i^	48/0	14/5	16/3	18/1	3.17^b^	2	.21	48/0	7/12	18/1	16/3	17.9^b^	2	**< .001**
*Cocaine hair analysis*^f^														
Cocaine_total_ pg/mg^h^	-	10.3 (29.2)	14.9 (32.2)	3.2 (9.9)	0.99^c^	2,54	.38	-	40.7 (76.1)	4.2 (8.2)°	3.2 (9.4)°	4.38^c^	2,54	**.02**
Cocaine pg/mg	-	8.2 (23.3)	11.4 (23.9)	2.5 (7.6)	0.98^c^	2,54	.38	-	31.7 (56.5)	3.1 (5.9)°	2.6 (7.9)°	4.81^c^	2,54	**.01**
Benzoylecgonine pg/mg	-	1.9 (5.5)	3.1 (7.6)	0.6 (1.9)	0.99^c^	2,54	.38	-	8.3 (19.6)	1.0 (2.2)	0.4 (1.2)	2.82^c^	2,54	.07
Cocaethylene pg/mg	-	1.0 (2.8)	0.9 (2.8)	0.3 (0.8)	0.45^c^	2,54	.64	-	1.2 (2.1)	0.3 (1.0)	0.7 (2.1)	1.02^c^	2,54	.37
Norcocaine pg/mg	-	0.2 (0.5)	0.4 (0.8)	0.1 (0.3)	1.11^c^	2,54	.34	-	0.6 (1.4)	0.1 (0.1)	0.1 (0.3)	2.81^c^	2,54	.07

Means and standard deviations. Significant p values (p < .05) are shown in bold. Sex and smoking are shown in frequency data.

^a^ ANOVA F-test (all groups, with significant Sidak post-hoc test vs. control group: ***p < .001; vs. cocaine increaser: °*p* < .05).

^b^ χ^2^-test (all groups) for frequency data.

^c^ ANOVA F-test (cocaine users only, with significant Sidak post-hoc test vs. cocaine increaser: °*p* < .05; °°°p < .001).

^d^ Average use during the last 6 months.

^e^ Since first cocaine use.

^f^ Hair samples were voluntary and data are deficient for 3 controls.

^h^ Cocaine_total_ (= Cocaine + Benzoylecgonine + Norcocaine) is a more robust procedure for discrimination between incorporation and contamination of hair [25].

^i^ Cut-off value for cocaine = 150 ng/ml.

^j^ Parameters at follow-up refer to the 1-year period between t1 and t2.

### Machiavellianism

**Fig 1:**
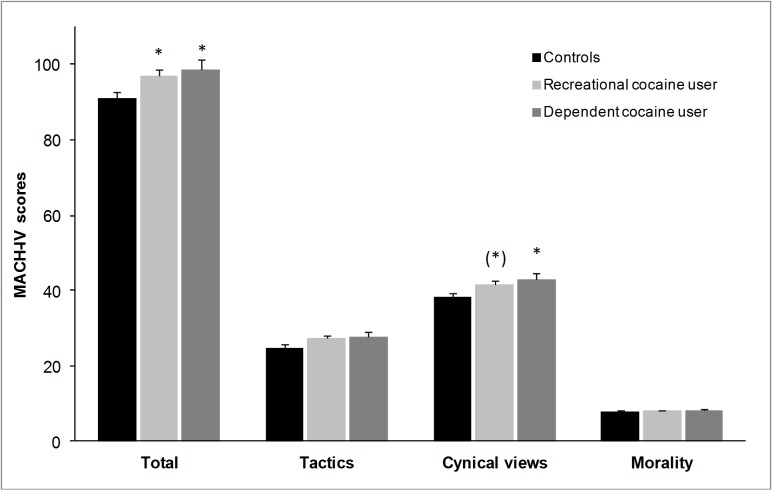
Mean scores and standard errors of means for the Machiavellianism-questionnaire MACH-IV at baseline (t1). [Legend: Values corrected for age and verbal IQ. Sidak post-hoc tests: ^(^*^)^p < .10; *p < .05. The values of one control, 2 RCU, and 2 DCU are missing.]

An ANCOVA (corrected for age and verbal IQ) showed significant group differences in the MACH-IV total scores (*F*(2,156) = 4.41, *p* < .05), whereby both RCU (*p* < .05, *d* = 0.43) and DCU (*p* < .05, *d* = 0.55) showed significantly higher MACH-IV total scores than controls but did not significantly differ from each other (*p* = .94, *d* = 0.12) (Fig 1).This effect was mainly driven by the subscale ‘cynical views’ (*F*(2,156) = 4.19, *p* < .05; *p*_*Controls vs*. *RCU*_ = .06, *d*_*Controls vs*. *RCU*_ = 0.40; *p*_*Controls vs*. *DCU*_ < .05, *d*_*Controls vs*. *DCU*_ = 0.55; *p*_*RCU vs*. *DCU*_ = .88, *d*_*RCU vs*. *DCU*_ = 0.15), but also the subscale ‘tactics’ showed a trend towards significant group differences (*F*(2,156) = 2.75, *p* < .10) with non-significant but moderate effect sizes for RCU (*p* = .12, *d* = 0.35) and DCU (*p* = .20, *d* = 0.42) compared to controls. The group differences in the subscale ‘morality’ were not significant (*F*(2,156) = 0.38, *p* = .69) and showed only slightly elevated values in both cocaine-using groups (*p*_*Controls vs*. *RCU*_ = .80, *d* = 0.14; *p*_*Controls vs*. *DCU*_ = .92, *d* = 0.13).

Notably, although the MACH-IV total scores effect size in DCU was larger than in RCU in comparison to the controls, Machiavellianism did not significantly correlate with dimensional cocaine use measures (Table 3). Importantly, if socio-economic status was introduced as an additional factor in a 2*2 ANOVA (median-split low [CHF 0–30'000] vs. high income [CHF >30'000] * CU vs. controls), the significant group differences in Machiavellianism remained, *F*(1, 157) = 6.29, *p* < .05), whereas the factor income was not significant, *F*(2, 159) = 1.11, *p* = .29).

**Table 3:** Correlations with Machiavellianism (MACH-IV total score) in cocaine users (CU) and stimulant-naïve controls at baseline (only Bonferroni-corrected p-values ≤.10 are shown).

	Controls + cocaine users (n = 166)		Only controls (n = 68)		Only cocaine users (n = 98)		Fisher's z Controls vs. cocaine users	
	r	p	r	p	r	p	z	P
**Temperament and Character Inventory (TCI)**								
*Cooperativeness*	**-0.55**	**< .001**	**-0.50**	**< .001**	**-0.55**	**< .001**	0.42	-
C1 Social acceptance vs. social intolerance	**-0.35**	**< .001**	-0.27	-	**-0.36**	**.005**	0.61	-
C2 Empathy vs. social disinterest	**-0.33**	**< .001**	**-0.38**	**.03**	-0.28	-	0.69	-
C3 Helpfulness vs. unhelpfulness	**-0.41**	**< .001**	**-0.42**	**.006**	**-0.37**	**.003**	0.36	-
C4 compassion vs. revengefulness	**-0.44**	**< .001**	**-0.40**	**.01**	**-0.47**	**< .001**	0.53	-
C5 Pure-hearted principles vs. self-advantage	**-0.41**	**< .001**	-0.29	-	**-0.44**	**< .001**	1.06	-
*Reward dependence*	**-0.34**	**< .001**	**-0.43**	**.005**	-0.23	-	1.45	-
RD1 Sentimentality vs. insensitivity	-0.21	-	-0.29	-	-0.13	-	1.03	-
RD3 Attachment vs. detachment	**-0.27**	**.009**	**-0.42**	**.007**	-0.14	-	1.88	-
RD4 Dependence vs. independence	**-0.26**	**.02**	-0.24	-	-0.21	-	0.19	-
*Self-directedness*	**-0.46**	**< .001**	**-0.45**	**.002**	**-0.44**	**< .001**	0.08	-
SD1 Responsibility vs. blaming	**-0.28**	**.005**	-0.33	-	-0.24	-	0.60	-
SD2 Purposefulness vs. goal-directed	**-0.37**	**< .001**	-0.34	-	**-0.36**	**.006**	0.14	-
SD3 Resourcefulness vs. apathy	**-0.33**	**< .001**	-0.25	-	**-0.40**	**.001**	1.03	-
SD4 Self-acceptance vs. self-striving	**-0.43**	**< .001**	**-0.44**	**.003**	**-0.40**	**.001**	0.30	-
SD5 Congruent second nature	**-0.24**	**.03**	-0.21	-	-0.22	-	0.06	-
**Multifaceted Empathy Test (MET)**								
Explicit emotional empathy	-0.12	-	-0.25	-	0.03	-	1.75	-
Implicit emotional empathy	-0.13	-	**-0.31**	**.03**	0.09	-	**2.52**	**.04**
Cognitive empathy	-0.05	-	-0.05	-	-0.07	-	0.12	-
**Movie for the Assessment of Social Cognition (MASC)**								
Total errors Theory-of-Mind	0.04	-	-0.02	-	0.10	-	0.74	-
No Theory-of-Mind	-0.03	-	-0.07	-	0.05	-	0.74	-
Insufficient Theory-of-Mind	0.02	-	-0.03	-	0.04	-	0.43	-
Too excessive Theory-of-Mind	0.08	-	0.06	-	0.10	-	0.25	-
**Reading the Mind in the Eyes Test (RMET)**								
Sum score	-0.11	-	-0.16	-	-0.05	-	0.68	-
**Games**								
Distribution Game Payoff B	**-0.29**	**< .001**	-0.24	-	**-0.31**	**.005**	0.46	-
Dictator Game Payoff B	-0.15	-	-0.07	-	-0.17	-	0.62	-
**Social Network Questionnaire (SNQ)**								
Total contacts	-0.03	-	-0.17	-	**0.20**	**.05**	**2.29**	**.02**
**Structured Clinical Interview (SCID-II)**								
*Cluster A*	**0.32**	**< .001**	**0.38**	**.03**	0.24	-	0.96	-
Paranoid personality disorder	**0.28**	**.005**	0.32	-	0.22	-	0.66	-
Schizoid personality disorder	**0.32**	**< .001**	**0.45**	**.002**	0.18	-	1.85	-
Schizotypal personality disorder	0.16	-	0.16	-	0.13	-	0.19	-
*Cluster B*	**0.52**	**< .001**	**0.44**	**.003**	**0.52**	**< .001**	0.57	-
Antisocial personality disorder	**0.25**	**.02**	0.31	-	0.16	-	0.97	-
Borderline personality disorder	**0.43**	**< .001**	0.28	-	**0.47**	**< .001**	1.36	-
Histrionic personality disorder	**0.32**	**< .001**	0.25	-	**0.32**	**.03**	0.47	-
Narcissistic personality disorder	**0.44**	**< .001**	**0.37**	**.03**	**0.44**	**< .001**	0.51	-
*Cluster C*	0.14	-	0.01	-	0.20	-	1.18	-
Avoidant personality disorder	0.15	-	0.10	-	0.19	-	0.56	-
Dependent personality disorder	0.05	-	-0.06	-	0.06	-	0.73	-
Obsessive-compulsive personality disorder	0.11	-	-0.02	-	0.20	-	1.36	-
*Other*	**0.37**	**< .001**	**0.36**	**.04**	**0.31**	**.04**	0.40	-
Depressive personality disorder	**0.25**	**.02**	0.26	-	0.21	-	0.32	-
Negativistic personality disorder	**0.36**	**< .001**	**0.37**	**.04**	0.29	-	0.55	-
**Cocaine use**^a^								
Times per week^b^					-0.03	-		
Grams per week^b^					-0.14	-		
Years of use					-0.01	-		
Max. dose (grams/day)					0.01	-		
Cumulative dose lifetime (grams)					-0.04	-		
*Cocaine hair analysis*								
Cocaine_total_ pg/mg					-0.12	-		
Cocaine pg/mg					-0.12	-		
Benzoylecgonine pg/mg					-0.11	-		
Cocaethylene pg/mg					-0.10	-		
Norcocaine pg/mg					-0.14	-		

Pearson’s product-moment correlations with Machiavellianism (MACH-IV total scores) and Fisher's z-tests. Significant p-values for correlations (p < .05, two-tailed) and z-tests (p < .05, two-tailed) are shown in bold. Only Bonferroni-corrected p-values ≤.10 are shown. P-Values were adjusted by Bonferroni correction according to the number of analyzed variables per test (TCI 16, MET 3, MASC 4, RMET 1, Games 2, SNQ 1, SCID-II 16, cocaine use measures 10). Controls n = 68, cocaine users n = 98. In MACH-IV, the values of one control and four cocaine users are missing; in the SCID-II, the values of one cocaine user are missing.

^a^ Cocaine use parameters were all ln-transformed because of the highly skewed distribution and the resulting deviation from the normal distribution (Shapiro-Wilk W < .001).

^b^ Average use during the last 6 months.

**Fig 2:**
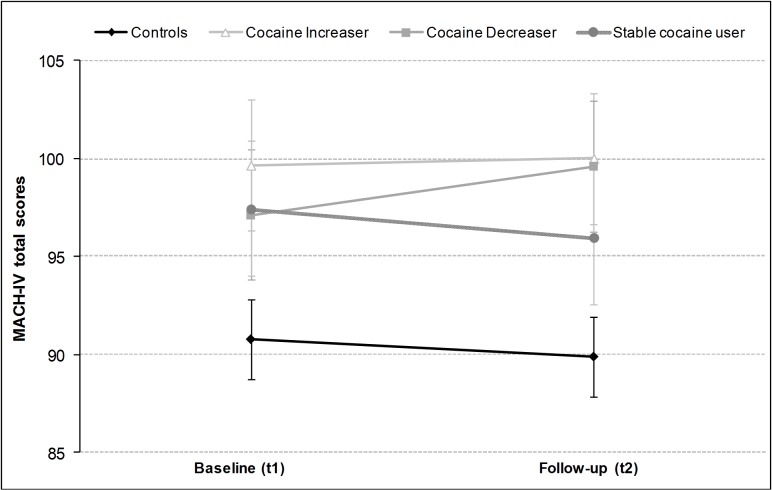
Mean scores and standard errors of means for the Machiavellianism-questionnaire MACH-IV at baseline (t1) and follow-up (t2). [Legend: 48 controls, 18 cocaine increaser, 18 cocaine decreaser, and 17 stable cocaine users.]

The longitudinal analysis using repeated measures ANCOVAs (covariates age and verbal IQ) showed a trend towards significance in the group effect (*F*(3,95) = 3.19, *p* = .09), but no-significant effect of time (*F*(1,95) = 1.21, *p* = .27) and also no significant group*time interaction for the MACH-IV total scores (*F*(3,95) = 0.55, *p* = .65) (Fig 2). An independent Student *t*-test between controls and a combined cocaine user group (increaser + decreaser + stable user) again confirmed the significant group differences at baseline (*t*(102) = 2.41, *p* < .05) and follow-up (*t*(100) = 3.02, *p* < .01) for the MACH-IV total score. Additionally, the test-retest reliability of the MACH-IV was relatively high in both controls (*r* = 0.79, *p* < .001) and cocaine users (*r* = 0.70, *p* < .001) as well as in the total sample (*r* = 0.75, *p* < .001).

### Machiavellianism-related TCI personality dimensions

Regarding the TCI scales, DCU showed significantly lower scores in the TCI main scales ‘cooperativeness’ and ‘self-directedness’ than the controls (Table 4), whereas RCU were intermediate between controls and DCU, showing a clear linear trend (cooperativeness: *p*_linear trend_ < .01, self-directedness *p*_linear trend_ < .001), but did not significantly differ from controls. With regard to the cooperativeness subscales, DCU reported significantly more social intolerance (C1) and more self-advantageous behavior (C5). Regarding self-directedness, only the subscale ‘resourcefulness vs. apathy’ (SD3) showed no significant difference between DCU and controls. Notably, DCU also differed significantly from RCU in self-directedness, which is mainly explained by significant differences in the subscales ‘responsibility’ (SD1), ‘purposefulness’ (SD2), and ‘congruent second nature’ (SD5).

**Table 4:** Measures of cooperativeness, reward dependence, and self-directedness in recreational and dependent cocaine users as well as stimulant-naïve controls.

	Stimulant-naïve controls (n = 68)	Recreational cocaine users (n = 68)	Dependent cocaine users (n = 30)	F	df,df_err_	p	p, Sidak post-hoc			Cohens' d		
							C-RCU	C-DCU	RCU-DCU	C-RCU	C-DCU	RCU-DCU
**Cooperativeness (TCI)**	32.54 (0.77)	30.71 (0.77)	28.75 (1.22)	3.71	2,157	**.03**	.26	**.03**	.45	.29	**.59**	.31
C1 Social acceptance vs. social intolerance	7.13 (0.17)	6.83 (0.17)	6.35 (0.27)	2.98	2,157	**.05**	.51	**.05**	.38	.21	**.54**	.33
C2 Empathy vs. social disinterest	5.46 (0.18)	5.42 (0.18)	4.84 (0.28)	1.84	2,157	.16	1.00	.19	.25	.03	.43	.40
C3 Helpfulness vs. unhelpfulness	6.21 (0.16)	5.81 (0.16)	5.62 (0.26)	2.54	2,157	.08	.22	.15	.90	.31	.45	.14
C4 compassion vs. revengefulness	7.01 (0.34)	6.56 (0.34)	6.67 (0.54)	.46	2,157	.63	.72	.93	1.00	.16	.12	.04
C5 Pure-hearted principles vs. self-advantage	6.72 (0.21)	6.10 (0.21)	5.26 (0.34)	6.97	2,157	**.001**	.11	**.001**	.11	.35	**.81**	.46
**Reward dependence (TCI)**	15.99 (0.49)	15.38 (0.49)	14.37 (0.77)	1.58	2,157	.21	.76	.22	.62	.15	.41	.25
RD1 Sentimentality vs. insensitivity	6.20 (0.27)	5.71 (0.27)	5.80 (0.42)	.90	2,157	.41	.48	.81	1.00	.23	.18	.04
RD3 Attachment vs. detachment	5.96 (0.23)	6.29 (0.23)	5.47 (0.36)	1.85	2,157	.16	.68	.58	.17	.17	.26	.43
RD4 Dependence vs. independence	3.82 (0.18)	3.38 (0.18)	3.10 (0.28)	2.94	2,157	.06	.21	.09	.79	.30	.48	.19
**Self-directedness (TCI)**	32.63 (0.87)	31.47 (0.88)	25.34 (1.38)	10.10	2,157	**< .001**	.73	**< .001**	**< .001**	.15	**.98**	**.82**
SD1 Responsibility vs. blaming	6.27 (0.24)	6.48 (0.24)	4.74 (0.38)	7.64	2,157	**< .001**	.91	**.003**	**< .001**	.10	**.75**	**.85**
SD2 Purposefulness vs. goal-directed	6.15 (0.22)	5.69 (0.22)	4.66 (0.35)	6.32	2,157	**.002**	.38	**.002**	**.05**	.25	**.80**	**.56**
SD3 Resourcefulness vs. apathy	3.87 (0.17)	3.93 (0.17)	3.17 (0.27)	2.94	2,157	.06	.99	.09	.06	.04	.50	.54
SD4 Self-acceptance vs. self-striving	7.58 (0.32)	6.95 (0.32)	5.99 (0.51)	3.57	2,157	**.03**	.43	**.03**	.30	.23	**.60**	.36
SD5 Congruent second nature	8.76 (0.28)	8.43 (0.28)	6.78 (0.45)	7.05	2,157	**.001**	.80	**< .001**	**.008**	.14	**.83**	**.69**

Means and standard errors. ANCOVA (all groups, corrected for age and verbal IQ). Significant p values (p < .05) are shown in bold. The values of one control, one RCU, and two DCU are missing. In contrast to a previous ZuCo^2^St-publication [18] that investigated TCI novelty seeking with the same sample, one RCU was excluded from the TCI analysis because too many relevant items were missing for the personality traits analyzed in this study.

### Correlation analyses

In the total sample (n = 166), Machiavellianism correlated negatively with the TCI scales ‘cooperativeness’, ‘reward dependence’, and ‘self-directedness’ (including all subscales), and with social decision-making in the Distribution Game, whereas it was positively linked to all cluster A, B, and ‘other’ PD symptoms according to *DSM-IV* (Table 3).

In cocaine users (n = 98), we found significant negative correlations for Machiavellianism with ‘cooperativeness’, ‘self-directedness’, and social decision-making, as well as a significant positive correlation for Machiavellianism with the cluster B PD.

Interestingly, higher Machiavellianism was associated with significantly lower implicit emotional empathy in the control group but not in cocaine users. Finally, cocaine users and controls significantly differed in their relationship between Machiavellianism and social network size: Among controls, higher Machiavellianism was linked to a slightly smaller social network, whereas a reverse pattern was detected among cocaine users.

### Machiavellianism at baseline and subsequent cocaine use

In order to test whether the level of Machiavellianism at baseline might predict cocaine use at the one-year follow-up, we applied three different analyses. Firstly, we classified all cocaine users as *low* or *high* according to a median split based on the MACH-IV total scores at baseline (cut-off score 96/97) [10, 38]. However, increaser (11 low/8 high), decreaser (10 low/8 high), and stable cocaine user (7 low/12 high) did not significantly differ regarding this ratio (χ^2^ (2) = 2.01, p = .37). Secondly, we compared MACH-IV total scores at baseline between cocaine increaser (mean = 99.52, SD = 3.40), cocaine decreaser (mean = 96.57, SD = 3.40), and stable cocaine users (mean = 98.20, SD = 3.47) by ANCOVA (corrected for age and verbal IQ), but could not find any significant group effect (*F*(2,51) = 0.19, *p* = .83). Thirdly, we correlated MACH-IV total scores at baseline with cocaine hair concentrations at follow-up (cocaine_total_ log) and the percental change of cocaine hair concentrations between baseline and follow-up (Δcocaine_total_ in %) but could neither detect any significant correlation within a consolidated cocaine user group (_*t2*_ = -.11, *p*_*t2*_ = .45; *r*_*Δ*_ = .09, *p*_*Δ*_ = .49), nor in the single groups of increaser (*r*_*t2*_ = -.14, *p*_*t2*_ = .56; *r*_*Δ*_ = .11, *p*_*Δ*_ = .65), decreaser (*r*_*t2*_ = -.29, *p*_*t2*_ = .31; *r*_*Δ*_ = -.09, *p*_*Δ*_ = .73), or stable cocaine users (*r*_*t2*_ = -.09, *p*_*t2*_ = .74; *r*_*Δ*_ = .26, *p*_*Δ*_ = .28).

## Discussion

The major finding of the present study is that both RCU and DCU exhibited a higher level of Machiavellianism than controls, reflecting a stronger tendency toward cynical views on human nature and–albeit less pronounced–toward interpersonal manipulation. Across one year, the elevated Machiavellianism was highly stable in cocaine users. Furthermore, DCU reported lower cooperativeness and self-directedness, which were both significantly correlated with higher Machiavellianism in a combined cocaine user sample (RCU+DCU). In general, controls and cocaine users showed similar relationships between Machiavellianism and socio-cognitive functioning and between Machiavellianism and PD. However, Machiavellianism correlated significantly negative with implicit emotional empathy in controls, whereas this correlation was not significant in cocaine users. Moreover, a negative correlation between Machiavellianism and social network size was displayed in controls, whereas the pattern was significantly reversed in cocaine users. Notably, although Machiavellianism was more prominent in DCU than in RCU, the absence of a significant correlation between Machiavellianism and indicators of cocaine use, as well as the high temporal stability of the MACH-IV scores, suggest that increased Machiavellianism might be a stable personality trait in cocaine users rather than a consequence of cocaine use. The latter has been proposed as a possible explanation for social cognition deficits and smaller social networks of cocaine users, as the cocaine dose has been found to correlate with socio-cognitive performance deficits [6]. Finally, MACH-IV scores of cocaine users at baseline did not predict their change of cocaine use within one year.

Remarkably, the MACH-IV score of 97.5 in our pooled cocaine user sample is highly similar to previously reported scores of 97.8 in abusers of ‘narcotics‘ [11] and 97.0 in methylphenidate abusers [39], indicating that substance use in general might be associated with a Machiavellian personality. Specifically, both cocaine users groups reported a pronounced cynical worldview and an increased use of deceit in interpersonal relationships; however, their morality scores did not differ from controls (it is important to note that morality was only rudimentarily measured with two items, wherein one item assessed morality in a very disputable manner by investigating participants’ opinions about euthanasia). Consequently, the pronounced Machiavellian thoughts and behaviors of cocaine users are probably explained by their unwillingness or inability to comply with the prevailing moral imperatives, although they are actually familiar with them.

In line with this, DCU displayed lower cooperativeness and self-directedness on the TCI, confirming previous findings in regular cocaine users and opiate-dependent individuals [40, 41]. In particular, the lower cooperativeness of DCU was mainly driven by two subscales indicating lower social tolerance and increased pursuit of self-advantage [16]. Furthermore, the subscales for self-directedness clearly reflected lower responsibility, purposefulness, resourcefulness, and self-control in DCU compared with RCU and controls [16]. Together with the elevated novelty seeking scores of our cocaine users [18], this temperament configuration can be called ‘antisocial‘, which is an essential part of the Machiavellian concept [8]. In summary, these findings are in accordance with our previous publications from the ZuCo^2^St sample in which altered social reward processing [42, 43], increased self-serving behavior [4], reduced emotional empathy, and more criminal offenses [6] were reported for chronic cocaine users.

McHoskey [9] stated that Machiavellianism is associated with various traits that may indicate personality dysfunction when they appear in extreme manifestations. Our findings support this assumption because we found significant positive correlations between Machiavellianism and SCID-II PD symptom scores in the total sample and both subsamples. In particular, the strongest link was found for cluster B PD, which is in line with the pronounced cluster B comorbidity reported in cocaine addicted individuals [7, 44].

The negative correlations between Machiavellianism and monetary payoffs for others in the social interaction tasks reflect the ecological validity of the MACH-IV with regard to prosocial behavior and confirm results reported by Bereczkei and Czibor [15]. These authors demonstrated in a healthy population that individuals with high levels of Machiavellianism maximize their profit in the public goods game. Notably, they also found similar negative relationships between Machiavellianism and the TCI scores self-directedness and cooperativeness as reported here [15].

By contrast, Machiavellianism did not correlate with emotion recognition and mental perspective taking, suggesting that Machiavellianism is independent of the ability to understand another person’s mental and emotional state. However, this finding differs from a previous report with participants who had all obtained at least a high school diploma, showing that the MACH-IV was correlated with self-reported empathy, alexithymia, and emotion recognition [14]. Interestingly, although we found a significant correlation between Machiavellianism and implicit emotional empathy in controls, this relationship was absent in cocaine users. The finding in controls is consistent with the literature, suggesting that high Machiavellianism is linked to diminished capabilities for emotional responses in terms of empathic concern (explicit) or arousal (implicit) [8].

Furthermore, higher Machiavellianism in cocaine users was related to having a broader social network, although their networks were in fact smaller than those of controls [6]. By contrast, controls showed the expected, although non-significant, negative link between Machiavellianism and social network size, confirming that Machiavellianism is inversely related to the self-reported importance of friendship [45]. We therefore hypothesize (I) that cocaine users establish rather low quality social relationships that are not affected by Machiavellian tendencies or (II) that high Machiavellianism is even helpful or popular in their peer environment.

Regarding the possibly underlying cortical structures, our findings fit well into the existing literature indicating a close relationship between chronic cocaine use and prefrontal dysfunctions [46] and between prefrontal dysfunctions and social behavior in general [1, 2] as well as Machiavellianism in particular [38, 47]. Given that Machiavellianism seems to be a stable and distinct trait in cocaine users, our data further support the notion of Ersche et al. [48] indicating that dysfunction of prefrontal control might potentially serve as a predictor for stimulant addiction or might even be considered an addiction endophenotype.

In conclusion, cocaine users showed significantly elevated and highly stable levels of Machiavellianism in general, while DCU also displayed lower cooperativeness and self-directedness measured with the TCI. Because cocaine use intensity and changing cocaine use patterns within one year did not affect Machiavellianism, and methylphenidate abuse has also been associated with higher Machiavellianism in a previous study from our lab [39], we propose that Machiavellianism might be a stable predisposition for stimulant use. Thus, in the future, the MACH-IV could be evaluated as a potential and simple tool for predicting the risk for stimulant addiction and abuse.

## Supporting information

S1 Dataset Supporting Information Dataset. (SAV)
